# Early-onset preeclampsia exposure and hospital outcomes of very preterm infants: a retrospective analysis of feeding intolerance and hospital morbidities

**DOI:** 10.3389/fped.2026.1847317

**Published:** 2026-06-25

**Authors:** Xinyue Li, Hui Zhang, Wenxin Dong, Tongyan Han

**Affiliations:** Department of Pediatrics, Peking University Third Hospital, Beijing, China

**Keywords:** feeding intolerance, infant, in-hospital morbidities, preeclampsia, premature

## Abstract

**Background:**

Maternal early-onset preeclampsia (EOPE) may affect gastrointestinal function in very preterm infants, but its independent association with feeding intolerance (FI) and in-hospital morbidities remains unclear.

**Methods:**

This retrospective 1:1 gestational age-matched cohort study included 87 very preterm infants (<32 weeks) exposed to EOPE and 87 controls born to normotensive mothers. The primary outcome was FI. The secondary outcomes included bronchopulmonary dysplasia (BPD), necrotizing enterocolitis (NEC), intraventricular hemorrhage (IVH), late-onset sepsis (LOS), retinopathy of prematurity (ROP), parenteral nutrition-associated cholestasis (PNAC), extrauterine growth restriction (EUGR), and mortality.

**Results:**

EOPE-exposed infants exhibited a significantly higher incidence of FI (77.0% vs. 41.4%, *P* < 0.001) and a prolonged time to full enteral feeding (13.0 vs. 9.5 days, *P* = 0.017). Multivariable logistic regression identified EOPE as an independent factor associated with FI (OR=2.290, 95% CI: 1.061–4.944, *P* = 0.035). Lower birth weight was also independently associated with an increased risk of FI.

**Conclusion:**

Maternal EOPE may be associated with an increased risk of FI in very preterm infants, underscoring the necessity for targeted feeding monitoring and nutritional support in this population.

## Introduction

Very preterm infants (gestational age <32 weeks) face increased risks of complications such as FI, NEC, and BPD due to organ immaturity, significantly impacting survival rates and long-term outcomes (1). As a prevalent gastrointestinal complication in preterm infants, FI manifests clinically with abdominal distension, gastric retention, and other symptoms, which not only prolong hospitalization and impair nutrient absorption but also correlate strongly with adverse neurodevelopmental outcomes (1). Notably, the risk of FI is significantly inversely correlated with advancing gestational age, whereas SGA infants present an up to sixfold increased risk of FI (2), suggesting that fetal growth restriction may profoundly disrupt gastrointestinal functional development (3).

EOPE, a placenta-derived pregnancy complication occurring before 34 weeks of gestation, is characterized by gestational hypertension accompanied by 24-hour urinary protein excretion ≥300 mg (4, 5). Placental insufficiency induced by EOPE triggers fetal chronic hypoxia, excessive release of inflammatory cytokines, and fetal growth restriction, which collectively contribute to pathophysiological alterations (6). Previous studies have confirmed that preterm infants born to EOPE mothers exhibit lower birth weights and suboptimal in-hospital growth trajectories (7); however, the feeding tolerance characteristics of this population during the very preterm period and their associations with in-hospital morbidities such as BPD and ROP remain inadequately elucidated (2, 8).

Although the association between EOPE and adverse neonatal outcomes has garnered increasing attention, most studies have focused on perinatal mortality, SGA incidence, and long-term neurodevelopmental sequelae, while systematic analyses of FI and other in-hospital morbidities, such as BPD, ROP, and IVH, in very preterm infants remain limited (2, 9). Notably, EOPE-exposed preterm infants generally exhibit lower mean gestational ages than their spontaneously preterm counterparts do. Previous studies often introduced bias due to inadequate control for gestational age confounders and a lack of standardized single-center clinical management protocols for comparative analysis. Although some evidence suggests that EOPE itself may not be an independent risk factor for FI (2), the confounding interaction between lower gestational age and SGA status could obscure the potential influence of EOPE (10), underscoring the necessity of gestational age-matched study designs to clarify its independent effects.

This study employed a 1:1 gestational age-matched cohort design (EOPE-exposed vs. spontaneous preterm groups) under standardized NICU protocols to systematically investigate the independent associations between maternal EOPE exposure and FI alongside in-hospital morbidities, including BPD, IVH, and ROP, in very preterm infants. By delineating the effects of EOPE exposure on hospitalization outcomes, this research provides a foundation for implementing individualized nutritional support strategies and enhancing complication surveillance in clinical management.

## Materials and methods

This retrospective cohort study included preterm infants with a gestational age < 32 weeks admitted to the NICU of Peking University Third Hospital between January 2021 and December 2023. The EOPE-exposed group comprised infants born to mothers diagnosed with EOPE, whereas the control group consisted of 1:1 gestational age-matched neonates delivered during the same period by normotensive mothers following spontaneous preterm labor. The inclusion criteria for both groups were as follows: (1) inborn preterm infants <32 weeks of gestation and (2) absence of congenital malformations or congenital infections. The study protocol was approved by the Medical Science Research Ethics Committee of Peking University Third Hospital (approval number: M20250585).

The nutrition management plan in this study aimed to optimize Total parenteral nutrition (TPN) and achieve total enteral feeding as early as possible. Specifically, parenteral nutrition was started within 24 h after birth, beginning with amino acids of 1.5 g/kg per day, which was increased by 0.5–1.0 g/kg per day with a maximum amount of 3.5 g/kg per day; beginning with fat of 0.5 g/kg per day, which was increased by 0.5–1.0 g/kg per day with a maximum amount of 3.0 g/kg per day; If there were no contraindications, enteral feeding (10–15 mL/kg per day) was started within 3 h after birth; and breast milk (with 35 mg of calcium, 15 mg of phosphate, 1.2 g of protein, and 67 kcal/100 mL) was preferred and increased at a rate of 10–15 mL/kg per day. When the amount of breast milk reached 80–100 mL/kg per day, a breast milk fortifier (BEBA FM85 in Germany: 1 g sachet in 20 mL of breast milk containing 100 mg of calcium, 60 mg of phosphate, 2.62 g of protein and 85 kcal/100 mL; or Similac HM Fortifier by Abbott of the United States: 0.9 g sachet in 25 mL of breast milk containing 146 mg of calcium, 81 mg of phosphate, 2.62 g of protein and 81 kcal/100 mL) was added, and half of the breast milk was fortified in the first 3–5 days and fully fortified later if tolerated. If the amount of breast milk was insufficient, the milk was supplemented with premature infant formula (PreNan, German Nestlé, containing 122 mg of calcium, 71 mg of phosphate, a protein content of 2.3 g and 84 kcal/100 mL). When enteral feeding reached 120 mL/kg per day, TPN was stopped (full enteral feeding goal was 160–180 mL/kg per day); vitamin D 700 IU was given orally every day beginning 8 days after birth.

The clinical data collected included maternal and neonatal demographic characteristics (including birth weight, gestational age, sex, singleton/multiple gestation, fetal distress, Apgar scores at 1 and 5 min, and umbilical cord blood pH), SGA status (defined as birth weight below the 10th percentile according to the Fenton and Kim intrauterine growth curves (11), and in-hospital outcomes such as FI and associated comorbidities during the hospitalization period.

The primary outcome was FI, which was defined as the presence of one or more of the following criteria: (1) abdominal distension (increase in abdominal circumference ≥2 cm); (2) gastric residuals exceeding 20% of the administered volume in two or more feedings; (3) vomiting; or (4) more than one episode of feeding interruption within a 24-hour period (12).

The secondary outcomes included other hospitalization comorbidities. BPD was diagnosed according to the National Institutes of Health criteria (13); NEC was considered when the stage was 2 or higher according to Bell's criteria (14); IVH and white matter injury were diagnosed by cranial ultrasound examination, and IVHwas graded by the Papile classification system (15); LOS required a blood culture positive for a causative pathogen obtained ≥ 72 h after birth and clinical sepsis symptoms (16); The diagnosis of ROP was made during screening by specialized ophthalmologists using indirect ophthalmoscopy (17); Parenteral nutrition-associated cholestasis(PNAC) was defined as a conjugated bilirubin level of ≥ 2 mg/dL (34.2 μmol/L) in infants receiving PN for≥14 days without an alternative etiology for hyperbilirubinemia (18); MBD was characterized by alkaline phosphatase (ALP) > 800 U/L, phosphorus <3.5 mg/dL (<1.1 mmol/L) and/or presence of radiological alterations: widening of the epiphyseal growth plate, loss of definition of the epiphysis/metaphysis calcification zone, and/or fractures (19); Gastrointestinal perforation required postoperative diagnosis by a pediatric surgeon; MRI was identified using ICD-10 codes indicative of a bowel obstruction secondary to inspissated meconium (20); Nosocomial pneumonia was defined as pneumonia occurring≥48 h after hospital admission in a patient (21); EUGR was defined as follows: (1) weight at discharge less than 10th centile (cross-sectional definition), (2) decrease of more than 1 SD since birth to the time of discharge home (longitudinal), and (3) “true” EUGR, either cross-sectional or longitudinal EUGR in non-SGA infants at birth (22). The secondary outcomes also included total oxygen use time and mortality rate.

### Statistical analysis

The data were analyzed via SPSS 26.0. Continuous variables are expressed as the mean ± standard deviation or median (interquartile range, IQR) on the basis of distribution normality, with group comparisons performed via Student's t test (parametric) or the Mann‒Whitney U test (nonparametric). Categorical variables are reported as frequencies (percentages), and differences between groups were assessed via the chi-square test or Fisher's exact test for small sample sizes. A two-tailed *P* value <0.05 was considered statistically significant. To identify independent factors associated with FI, multivariable logistic regression analysis was performed. All variables with *P* < 0.2 in the univariate analysis (including EOPE exposure status, birth weight, fetal distress, small-for-gestational-age status, umbilical cord blood pH, and Apgar score at 1 min) were included in the initial model. A backward stepwise elimination method based on the Wald statistic, with a removal criterion of *α* = 0.10, was then applied. Covariates that did not meet the retention threshold were subsequently excluded from the final model.

## Results

### Trial population

**Figure 1:**
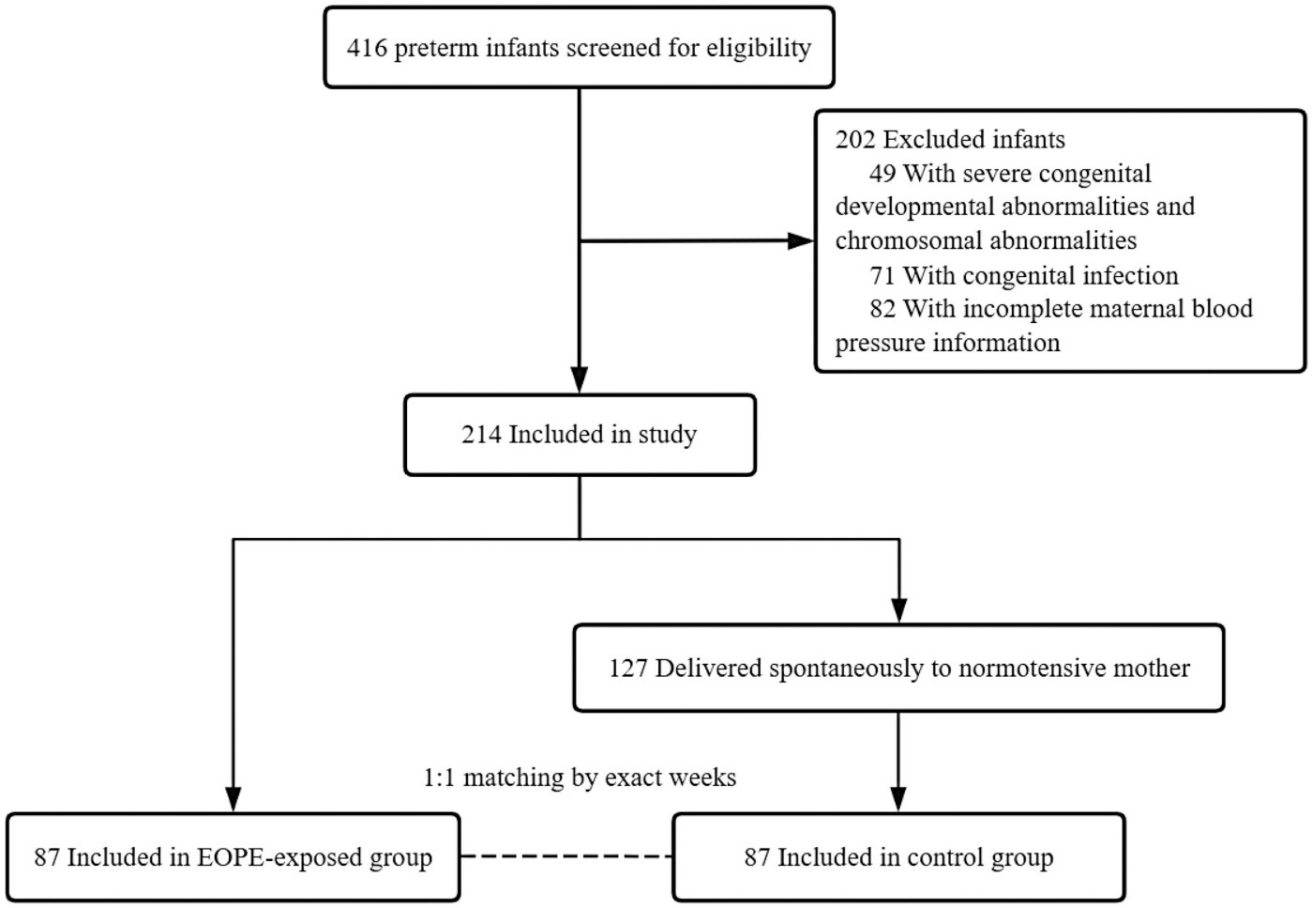
Patient recruitment and randomization EOPE, early-onset preeclampsia.

During the study period (January 2021-December 2023), a total of 416 preterm infants were assessed for eligibility (Figure 1). Of these, 202 infants (48.6%) were excluded because they did not meet the inclusion criteria. The final cohort comprised 87 infants in the EOPE-exposed group and 87 gestational age-matched controls (1:1 matching by exact weeks). Both groups had a median gestational age of 30.1 weeks. However, infants in the EOPE group presented significantly lower birth weights than did the controls (1240 g vs. 1380 g, *P* < 0.001), along with higher rates of fetal distress (20.7% vs. 8.0%, *P* = 0.017) and SGA (17.2% vs. 3.4%, *P* = 0.003) (Table 1).

**Table 1:** Infant baseline demographic and clinical characteristics.

Characteristic	EOPE-exposed group (*n* = 87)	Control group (*n* = 87)	Z/*χ*^2^	*P*
Gestational age at birth, median (IQR),wk	30.1 (28.4–31.1)	30.1 (28.2–31.1)	−0.078	0.938
Birth weight, median (IQR),g	1240.0 (940.0–1430.0)	1380.0 (1150.0–1595.0)	−4.743	＜0.001
Female, No.(%)	45 (51.7)	49 (56.3)	0.370	0.543
Fetal distress, No.(%)	18 (20.7)	7 (8.0)	5.652	0.017
Small-for-gestational-age, No.(%)	15 (17.2)	3 (3.4)	8.923	0.003
Umbilical cord blood pH, median (IQR)	7.4 (7.3–7.4)	7.4 (7.3–7.4)	−1.462	0.144
Apgar score at 1 min, median (IQR)	10.0 (8.0–10.0)	10.0 (9.0–10.0)	−1.897	0.058
Apgar score at 5 min, median (IQR)	10.0 (9.0–10.0)	10.0 (10.0–10.0)	−0.806	0.420
Apgar score at 10 min, median (IQR)	10.0 (9.0–10.0)	10.0 (10.0–10.0)	−0.720	0.472

Data are presented as median [interquartile range (IQR)], number (%), or mean ± standard deviation (SD) where specified. Group comparisons used Mann–Whitney U test (Z-values) for continuous variables and χ^2^ test for categorical variables.

EOPE, early-onset preeclampsia; wk, weeks; g, grams; pH: potential of hydrogen; min: minutes.

### Primary outcome

The incidence of FI was significantly greater in the EOPE-exposed group than in the control group (77.0% vs. 41.4%, *χ*^2^ = 22.865, *P* < 0.001). Additionally, the median time to achieve full enteral feeding was prolonged in the EOPE group (13.0 d vs. 9.5 d, Z = −2.395, *P* = 0.017). In contrast, the EOPE group demonstrated a shorter mean duration to regain birth weight (10.8 ± 2.8 d vs. 12.7 ± 4.2 d, Z = −2.489, *P* = 0.015) than did the control group (Table 2). Multivariable logistic regression analysis was further performed to identify independent factors associated with FI. After backward stepwise selection, maternal EOPE and birth weight remained in the final model, whereas fetal distress, SGA, umbilical cord blood pH, and Apgar score at 1 min were excluded from the final model (*P* ≥ 0.10). EOPE was independently associated with a significantly increased risk of FI (OR=2.290, 95% CI: 1.061–4.944, *P* = 0.035). Lower birth weight (per 100 g increase) was also identified as an independent protective factor for FI (OR=0.749, 95% CI: 0.649–0.863, *P* < 0.001). Umbilical cord blood pH (per 0.1 unit increase) did not reach statistical significance (OR=0.535, 95% CI: 0.267–1.070, *P* = 0.077) (Table 3).

**Table 2:** Comparison of primary outcomes between two groups.

Characteristic	EOPE-exposed group (*n* = 87)	Control group (*n* = 87)	Z/χ2	P
Feeding intolerance, No.(%)	67 (77.0)	36 (41.4)	22.865	＜0.001
Time to achieve full enteral feeding, median (IQR), d	13.0 (9.8–18.3)	9.5 (7.0–14.3)	−2.395	0.017
Time to regain birth weight, mean (SD), d	10.8 (2.8)	12.7 (4.2)	−2.489	0.015

Data presented as number (%), median (IQR), or mean ± SD. Statistical comparisons used χ^2^ test for categorical data and Mann–Whitney U test (Z-values) for continuous variables.

FI, feeding intolerance; d, days.

**Table 3:** Multivariable logistic regression analysis of factors associated with feeding intolerance in very preterm infants.

Predictors	*β*-value	OR	95%CI	*P*
Maternal early-onset preeclampsia (yes vs. no)	0.828	2.290	1.061, 4.944	0.035
Birth weight, per 100 g increase	−0.289	0.749	0.649, 0.863	＜0.001
Umbilical cord blood pH, per 0.1 unit increase	−0.626	0.535	0.267, 1.070	0.077

The covariates included in the binary logistic regression model were: EOPE exposure status, birth weight, fetal distress, SGA, umbilical cord blood pH, and Apgar score at 1 min. The backward Wald method (removal criterion *α* = 0.10) was used for variable selection, in which fetal distress, SGA, and Apgar score at 1 min were removed (*P* ≥ 0.10) and excluded from the final model. Odds ratios for continuous variables are presented per 100 g increase in birth weight and per 0.1 unit increase in cord blood pH. CI, confidence interval; OR, odds ratio.

### Secondary outcomes

The incidence of MRI was greater in the EOPE-exposed group than in the control group (9.2% vs. 1.1%, *P* = 0.034). Moreover, the incidence rates of PNAC (20.0% vs. 1.2%, *χ*^2^ = 15.489, *P* < 0.001) and EUGR (61.3% vs. 23.8%, *χ*^2^ = 22.979, *P* < 0.001) were higher in the EOPE-exposed group than in the control group. Additionally, the proportions of patients in the EOPE-exposed group with stage 2 or higher ROP (21.3% vs. 9.4%, *χ*^2^ = 4.312, *P* = 0.038) and LOS (28.7% vs. 16.1%, *χ*^2^ = 3.999, *P* = 0.046) were greater than those in the control group. The EOPE-exposed group required longer invasive respiratory support [33.5 (0.0–72.0) vs. 0.0 (0.0–5.0) hours, t = −3.434; *P* = 0.001], but there was no statistically significant difference in total oxygen use time between the two groups. The other aspects, including BPD, severe IVH, NEC, MBD, gastrointestinal perforation, nosocomial pneumonia and the mortality rate, did not significantly differ between the two groups (Table 4).

**Table 4:** Comparison of the secondary outcomes between two groups.

Characteristic	EOPE-exposed group (*n* = 87)	Control group (*n* = 87)	Z/χ^2^	*P*
BPD grade in survivors at 36 wk, No./total (%)				
None	55/75 (73.3)^b^	72/84 (85.7)^c^		0.226^a^
1	14/75 (18.7)^b^	8/84 (9.5)^c^		
2	5/75 (6.7)^b^	3/84 (3.6)^c^		
3	1/75 (1.3)^b^	1/84 (1.2)^c^		
IVH(Grade III or IV), No.(%)	13 (14.9)	8 (9.2)	1.354	0.245
NEC, No. (%)	1 (1.1)	3 (3.4)		0.621^a^
Gastrointestinal perforation, No.(%)	6 (6.9)	1 (1.1)		0.117^a^
MRI, No. (%)	8 (9.2)	1 (1.1)		0.034^a^
Nosocomial pneumonia, No. (%)	11 (12.6)	17 (19.5)	1.532	0.216
LOS, No. (%)	25 (28.7)	14 (16.1)	3.999	0.046
MBD, No./total (%)	17/75 (22.7)^b^	13/84 (15.5)^c^	1.338	0.247
PNAC, No./total (%)	15/75 (20.0)^b^	1/84 (1.2)^c^	15.489	＜0.001
ROP(stage≥2), No./total (%)	16/75 (21.3)^b^	8/84 (9.4)^c^	4.312	0.038
EUGR, No./total (%)	46/75 (61.3)^b^	20/84 (23.8)^c^	22.979	＜0.001
In-hospital mortality, No. (%)	2 (2.3)	0 (0.0)		0.497^a^
Total duration of IMV, median (IQR), h	33.5 (0.0–72.0)	0.0 (0.0–5.0)	−3.434	0.001
Total duration of NIV, median (IQR), d	21.5 (12.3–38)	23.5 (10.8–40.0)	−0.067	0.946
Duration of supplemental oxygen, median (IQR), d	24.5 (15.0–41.3)	26.0 (12.5–40.8)	−0.096	0.923

BPD, bronchopulmonary dysplasia; IVH, intraventricular hemorrhage; NEC, necrotizing enterocolitis; MRI, meconium-related ileus; LOS, late-onset sepsis; MBD, metabolic bone disease; PNAC, parenteral nutrition-associated cholestasis; ROP, retinopathy of prematurity; EUGR, extrauterine growth restriction; IMV, invasive mechanical ventilation; h, hours; NIV, non-invasive ventilation; d: days.

Data presented as number/total (%) or median (IQR). Statistical tests: χ2/Fisher’s exact test for categorical variables, Mann–Whitney U test (Z-values) for continuous variables.

a Fisher’s exact test.

b In the EOPE group (*n* = 87), 2 infants died, 8 were transferred, and 2 discontinued treatment, 75 survivors to discharge.

c In the Control group (*n* = 87), 3 infants were transferred, and 84 survivors to discharge.

## Discussion

This study demonstrated that preterm infants exposed to maternal EOPE are at a significantly higher risk for FI, SGA status, and related comorbidities than matched controls. The EOPE-exposed group exhibited significantly higher rates of FI and SGA, along with a prolonged time to achieve full enteral feeding. Multivariable logistic regression further confirmed that EOPE was independently associated with FI (OR=2.290, 95% CI: 1.061–4.944, *P* = 0.035) after adjusting for potential confounders. Previous studies have shown that maternal EOPE leads to placental hypoperfusion (23). Such hypoperfusion may trigger a compensatory reduction in fetal intestinal blood flow, thereby impairing intestinal maturation and motility. Consequently, EOPE-exposed infants had a higher incidence of MRI. Importantly, despite a higher incidence of FI and a delay in achieving full enteral feeding, this group paradoxically demonstrated a shorter time to regain birth weight.

This apparent paradox may reflect more aggressive combined enteral-parenteral nutritional support in SGA infants. Studies indicate that SGA infants receive significantly greater cumulative amino acid and lipid emulsion intake during the second postnatal week than non-SGA infants do (24). While accelerating weight recovery, this high nutritional load may exacerbate the risk of cholestasis. Our analysis confirmed a substantially higher incidence of PNAC in EOPE-exposed infants. SGA preterm infants are particularly prone to this condition because of long-term parenteral nutrition and the cumulative intake of amino acids and lipid emulsions. Furthermore, elevated liver shear wave elastography (SWE) values in SGA preterm infants are associated with cholestasis, suggesting that liver dysfunction may further affect intestinal nutrient absorption (25). PNAC can cause deficiencies in fat-soluble vitamins and reduce energy intake, which further increases the risk of EUGR. Our study revealed that preterm infants exposed to EOPE presented a significantly greater incidence of EUGR than controls did. Notably, a recent study in preterm infants born at <34 weeks gestation identified low birth weight, cholestasis, and neonatal sepsis as independent risk factors for EUGR (26). Consistent with these findings, our study demonstrated that EOPE-exposed infants had significantly lower birth weight, and higher incidences of PNAC and LOS compared to controls. These converging lines of evidence explain why EOPE-exposed very preterm infants are at a substantially higher risk of developing EUGR during hospitalization. Thus, EOPE exposure may not only be associated with lower birth weight, but may also exacerbate postnatal growth failure by increasing the risks of cholestasis and infection.

Our research revealed that preterm infants exposed to EOPE had longer durations of invasive mechanical ventilation and an increased incidence of stage 2 or higher ROP. Studies in preterm infants born at <34 weeks of gestation also found that the EOPE-exposed group had a longer duration of invasive mechanical ventilation, which is consistent with our findings (27). This may be related to lower birth weight and a higher incidence of FI in this population. Although no direct evidence supports a causal relationship between EOPE and ROP, invasive mechanical ventilation has been confirmed as an independent risk factor for ROP in preterm infants (28). Furthermore, no significant differences were observed between the groups regarding the incidence rates of BPD, MBD, NEC, gastrointestinal perforation, severe IVH or mortality. Existing literature indicates that maternal preeclampsia itself is not a significant risk factor for BPD (29). However, preeclampsia has been associated with an increased incidence and mortality of NEC and gastrointestinal perforation in preterm infants (30, 31). The literature has not directly explored the risk of MBD in preterm infants with preeclampsia. However, SGA preterm infants may have an increased risk of MBD indirectly because of factors such as prolonged parenteral nutrition, vitamin D deficiency, and calcium‒phosphorus metabolism disorders (24, 25). Similar to our results, the incidence of IVH was higher but did not reach statistical significance in preterm infants exposed to EOPE (27). This association may stem from preeclampsia-induced fetal hypoxia and hemodynamic instability resulting from placental dysfunction, as unstable cerebral blood flow is a recognized precipitant of severe IVH in preterm infants (32).

This study employed a gestational age-matched design to isolate the effects of EOPE from those of prematurity. The standardized nutritional and respiratory protocols within a single-center cohort enhanced internal validity, minimizing variability in care practices that often obscure associations between placental pathology and outcomes such as FI or MBD. However, the retrospective design may introduce the potential for residual confounding from unmeasured factors, such as maternal medication adherence or genetics. Although the sample size was adequate for primary outcomes, the investigation of rare complications (e.g., NEC and mortality) warrants larger multicenter studies for validation. The lack of long-term neurodevelopmental data also constrains the assessment of the lifelong impact of EUGR. Future research should further refine the distinctions among EOPE, preterm preeclampsia, and term preeclampsia to better characterize their respective pathophysiological roles and clinical implications. Despite these limitations, the study successfully disentangled EOPE's biological effects from prematurity-related risks, providing actionable insights for clinical prioritization in this high-risk population.

## Conclusion

In this gestational age-matched retrospective cohort study, exposure to EOPE was independently associated with an increased risk of FI and multiple in-hospital morbidities in very preterm infants, including PNAC, EUGR, MRI, LOS, and stage 2 or higher ROP. Multivariable logistic regression analysis revealed that EOPE was independently associated with FI (OR=2.290, 95% CI: 1.061–4.944, *P* = 0.035). However, due to the retrospective single-center design and the potential for residual confounding, causality cannot be established. These findings warrant validation through prospective, multicenter studies. EOPE-exposed very preterm infants should be recognized as a distinct high-risk subgroup, warranting proactive feeding monitoring, tailored nutritional support, and early screening for cholestasis and extrauterine growth restriction.

## Data Availability

The original contributions presented in the study are included in the article/Supplementary Material, further inquiries can be directed to the corresponding author.
